# Determinants Associated With the Risk of Emergency Department Visits Among Patients Receiving Integrated Home Care Services: A 6-Year Retrospective Observational Study in a Large Italian Region

**DOI:** 10.34172/ijhpm.2020.79

**Published:** 2020-06-07

**Authors:** Sara Campagna, Alberto Borraccino, Gianfranco Politano, Alfredo Benso, Marco Dalmasso, Valerio Dimonte, Maria Michela Gianino

**Affiliations:** ^1^Department of Public Health and Paediatrics, Università di Torino, Torino, Italy.; ^2^Department of Control and Computer Engineering, Politecnico di Torino, Torino, Italy.; ^3^Epidemiology Unit, Local Health Unit TO3, Grugliasco (TO), Piedmont, Italy.

**Keywords:** Emergency Department visits, Integrated Home Care, Administrative Health Data, Risk Factors, Retrospective Study Italy, Ethical Issues

## Abstract

**Background:** Allowing patients to remain at home and decreasing the number of unnecessary emergency room visits have become important policy goals in modern healthcare systems. However, the lack of available literature makes it critical to identify determinants that could be associated with increased emergency department (ED) visits in patients receiving integrated home care (IHC).

**Methods:** A retrospective observational study was carried out in a large Italian region among patients with at least one IHC event between January 1, 2012 and December 31, 2017. IHC is administered from 8 am to 8 pm by a team of physicians, nurses, and other professionals as needed based on the patient’s health conditions. A clinical record is opened at the time a patient is enrolled in IHC and closed after the last service is provided. Every such clinical record was defined as an IHC event, and only ED visits that occurred during IHC events were considered. Sociodemographic, clinical and IHC variables were collected. A multivariate, stepwise logistic analysis was then performed, using likelihood of ED visit as a dependent variable.

**Results:** A total of 29209 ED visits were recorded during the 66433 IHC events that took place during the observation period. There was an increased risk of ED visits in males (odds ratio [OR]=1.29), younger patients, those with a family caregiver (OR=1.13), and those with a higher number of cohabitant family members. Long travel distance from patients’ residence to the ED reduced the risk of ED visits. The risk of ED visits was higher when patients were referred to IHC by hospitals or residential facilities, compared to referrals by general practitioners. IHC events involving patients with neoplasms (OR=1.91) showed the highest risk of ED visits.

**Conclusion:** Evidence of sociodemographic and clinical determinants of ED visits may offer IHC service providers a useful perspective to implement intervention programmes based on appropriate individual care plans and broad-based client assessment.

## Introduction

Formal home care is a rapidly growing area and a critical component of an effective healthcare system.^1^ Formal home care services are different from informal home care services (those provided by family, friends, or neighbours). Formal healthcare is defined as a modality of healthcare and social assistance that is provided to patients in their home by healthcare providers or volunteer organisations.^2^ These services are meant to meet a range of needs, such as short-term care for acute conditions, recovery from surgery, long-term care for people with chronic conditions and disabilities, and other specific needs, such as end-of-life care, rehabilitation, and support for family caregivers.^2^

The Italian National Healthcare System guarantees that people who are not self-sufficient or are part of a vulnerable population due to socioeconomic or health-related factors can receive individualised, home-based care that corresponds to their health needs. The intensity, complexity, and duration of such care is classified as follows: (*i*) home care cure, aimed at people who require the occasional or scheduled services (weekly or monthly) of a general practitioner or nurse; and (*ii*) integrated home care (IHC), aimed at people who require more constant care. IHC consists of a variety of services to meet a range of needs, from short-term care for those recovering from surgery or acute medical conditions, to long-term care and rehabilitation support to allow people with chronic conditions to continue living in the community.^3^ Before patients are enrolled in IHC, a multidimensional assessment is carried out and a formal individual care plan is created. The purpose of individual care plans is to map needed care and ensure patients receive this care in their home, thus reducing the number of hospital admissions and unnecessary visits to the emergency department (ED). In the Italian healthcare system, home care cure and IHC can only be provided to patients in their home; they cannot be provided at residential facilities or residential communities.

Much of the recent literature has focused on the occurrence and appropriateness of ED visits among patients from residential, full-time care facilities^4–7^; to the authors’ knowledge, few studies have analysed the issue of ED visits from the home care perspective.^8,9^ For patients receiving IHC, an ED visit does not necessarily represent a negative outcome if the visit is deemed appropriate. However, when the patient can avoid or prevent an ED visit by receiving the correct treatment directly in the home care setting, an ED visit should be considered a negative health outcome. Although it is difficult to determine the appropriateness of an ED visit, it would be of great help to identify the socio-demographic and clinical characteristics that lead to an excess of avoidable ED visits.

 The aim of this study was to assess possible determinants associated with the risk of ED visits in patients receiving IHC. Analyses were run to test the hypothesis that ED visits could be partly explained by socio-demographic, clinical and IHC variables.

## Key Messages

**Implications for policy makers**

- This study deals with an issue that is common to all modern healthcare systems, which are called to provide continuity of care and appropriate assistance for the health needs of specific populations.
- Patient-centred healthcare systems should ensure that citizens receive formal home care services so that people of all ages can receive needed care at home rather than in a hospital or long-term care facility. This enables patients to remain at home and reduces the number of unnecessary emergency department (ED) visits.
- Evidence of socio-demographic and clinical determinants of ED visits may offer integrated home care (IHC) service providers a useful perspective to implement intervention programmes based on appropriate individual care plans and broad-based client assessment.

**Implications for public**

This work contributes to the body of literature focused on integrated home care (IHC) services, which is an alternative to residing in healthcare facilities and is potentially able to reduce patient discomfort due to isolation and unfamiliar surroundings. Through IHC, patients can receive appropriate treatment and reduce emergency department (ED) visits. Unfortunately, there is still a lack of studies that analyse the determinants that could be associated with increased ED visits by these patients. The results that emerged in this study can actively increase the available knowledge in this field.

## Methods

### Study Design and Setting

A retrospective observational study was carried out in the Piedmont Region, the second largest region of Italy, which has a population of more than 4 million inhabitants over an area of 25387 km^2^.^10^

###  Participants 

 Official, compulsory electronic medical records were used to construct a population-based cohort of patients with at least one IHC event between January 1, 2012 and December 31, 2017. Before patients are enrolled in IHC, a multidimensional assessment is carried out. After enrolment, IHC is administered from 8 am to 8 pm by a team of physicians, nurses, and other professionals as needed based on the patient’s health conditions. A clinical record is opened at the time a patient is enrolled in IHC and closed after the last service is provided. Every such clinical record was defined as an IHC event, and IHC events represented the unit of analysis. As such, there may have been some patients with 2 or more IHC events during the observation period. IHC events, rather than patients, were then linked to available ministerial ED records to identify ED visits that occurred during IHC events.

###  Data Sources

 IHC events, patient characteristics, and ED visits were collected by merging 2 different information sources: the Sistema Informativo Assistenza Domiciliare (SIAD) database (that is the official Italian national information monitoring system for home care services), and the Italian National Information System for ED use database.

Following this pre-processing of data collection and source merging, we had created (*i*) a matrix of sequential IHC events that occurred during the observation period, linked to one or more ED visits, and (*ii*) a matrix of patients with at least one IHC event, regardless of whether he/she had corresponding ED visits. The latter matrix, representing only IHC events, was used to test the existence of confounding cluster effects.

###  Variables

The socio-demographic and clinical variables in analyses refer to IHC events, not patients, as patients can vary across IHC events. Socio-demographic variables were gender, age, presence or absence of a non-family caregiver, number of cohabitant family members (living alone, 1, 2, 3, 4, >4 members), and travel distance in minutes to the closest ED (≤5, 6-8, <20 minutes). Age of patients at the time of the IHC event was collected and stratified into 6 age categories: paediatric/developmental ages (≤18 years); adults (19-65, 66-80 years), and older adults, which was further split (81-90, 91-100, >100 years). Travel distance to the closest ED was obtained from the National Agency for Territorial Cohesion, which classifies areas of residence based on the average distance to the nearest territorial ED.^11^ Clinical variables included prevalent disorder at IHC enrolment and pathology registered at the ED visit (as coded from International Classification of Diseases version 9 codes-CM, ICD-9-CM), which was taken from the SIAD database.

 IHC variables included the proponent of referral to IHC (general practitioner, hospital, residential facility/other settings) and duration of IHC event (number of days from enrolment to IHC last service delivered or patient death), classified in 100-day categories. To better describe ED visits, the following variables were also included time of arrival in ED (6 am-2 pm, 2 pm-8 pm, 8 pm-12 pm, 12 pm-6 am) and destination after discharge from ED visit (home following treatment in ED, admitted to hospital, died in ED, home with no treatment needed in ED, residential facilities).

###  Data Analysis 

Data was analysed from a descriptive perspective, and further risk and regression analyses were performed to better uncover the roles of investigated determinants, and to test for the presence of possible confounding effects. All analyses were performed by using R.^12^ In particular, we took advantage of the following packages: EpiR,^13^ Complex Heatmap,^14^ and GLM, on top of which we coded a computational pipeline that handled all data pre-processing steps and provided detailed results. Contingency-table, chi-square test, and Fisher exact test results are shown in Supplementary file 1.

The R pipeline provided a complete report to assess the risk of ED visits per stratum and a descriptive analysis, which was useful to assess the overall population distribution across strata. A risk analysis was performed to highlight which determinants increased the risk of ED visits during the IHC events in the observation period. The frequency of ED visits for each investigated determinant’s strata was summarised into a multiclass contingency table. Then, for each contingency table, the first stratum of interest was chosen as reference, and all the other strata were tested against it, thus resulting in comparable risks, thanks to their common reference stratum (see Gianino et al^15^ for more statistical details). Each contingency table was tested against the chi-square test and (for low numerosity strata determinant’s) the Fisher exact test to address non-randomness, and the significance of the risk analysis was also computed. A further, multivariate, stepwise logistic regression analysis was performed, using likelihood of ED visit as the dependent variable, and mutually adjusted for each of the other independent variables to take into account the presence of confounding effects. Risk of ED visit was reported as odds ratios (ORs) and corresponding 95% confidence intervals (CIs), with a significance level at *P* < .05. To highlight possible differences in the study sample due to recurring IHC events, a second stepwise logistic regression analysis was performed that was restricted to the first IHC event for each patient (first-appearances). Due to differences in the population, both Akaike information criterion and Bayesian information criterion are not comparable, and an analysis of variance test cannot be performed, since the fitted models have different degrees of freedom. Thus, to assess if any difference was present, we used both the fitted models to predict the probability of ED visits in a common population. We used both populations (full study sample and first-appearances) as test sets and tested the distribution of predicted probabilities.

A Disease/Diagnosis correlation analysis was performed using the Complex Heatmap package.^14^ The resulting map reported the different conditions under which the IHC was provided in rows and the Disease/Diagnosis registered for each ED visit in columns. Heatmap reported the occurrence of the pathology registered at the ED visit in different colours shades under that specific IHC.

## Results

During the 6-year observation period, a total of 44431 patients who received one or more IHC events had an ED visit recorded (Table 1). The mean of IHC events and ED visits per patient was 1.54 and 0.66, respectively.

**Table 1:** Baseline Characteristics of Patients Receiving IHC Services (N = 44431) Who Visited the ED, Between the Year 2012 and the Year 2017

**At Least 1 IHC and 1 ED Visit **	**N**	**Percent**
Gender		
Female	23788	53.5
Male	20643	46.5
Age groups (y)		
≤18	448	1.0
19-65	8512	19.2
66-80	14977	33.7
81-90	15317	34.5
91-100	4986	11.2
>100	191	0.5

Abbreviations: IHC, integrated home care; ED, emergency department. IHC events and ED visits per person during the 6 years observation were respectively 1.5 (SD = 1.28) and 0.66 (SD = 1.34).

###  Integrated Home Care Events

Results of the second stepwise logistic regression analysis, which compare 2 sets: (*i*) full study sample, and (*ii*) first-appearances (see Data Analysis section), showed that the 2 models performed differently (*t* student, *P* < .001) during the 6-year observation with a slightly more conservative estimation (lower probability of ED visit) in the first-appearances subset. This difference, although significant, was not very large in terms of probability and suggests that, for the few highly recurring cases, it may be worth considering a different treatment to reduce the overall probability of ED visits.

A total of 66433 IHC events were recorded, 52.7% of which involved female patients aged 66-90 years (Table 2). In 75.3% of IHC events, the patient was living alone, at a distance of less than 20 minutes from an ED. In 93.1% of IHC events, the patient had a family caregiver, and 69.2% of patients were referred to IHC by a general practitioner.

**Table 2:** Sociodemographic, Clinical and IHC Variables, Adjusted ORs and 95% CI of Registered IHC Events Having ED Visits Between 2012 and 2017

	**IHC Events (N = 66433)**		**ED Visits (N = 29209)**		**ED Visits/IHC Events**	**Adjusted OR** ^a^	**95% CI**
	**No.**	**%**	**No.**	**%**	**Per 100 Events**		
Gender							
Female subjects	35032	52.7	14163	48.5	40.4	1	
Male subjects	31401	47.3	15046	51.5	47.9	1.29*	1.24-1.33
Caregiver							
Non-family	4591	6.9	1871	6.4	40.8	1	
Family	61842	93.1	27338	93.6	44.2	1.13*	1.06-1.22
Age (y)							
≤18	1236	1.9	769	2.6	62.2	1	
19-65	14027	21.1	6840	23.4	48.8	0.82*	0.72-0.93
66-80	22757	34.3	10805	37.0	47.5	0.83*	0.73-0.95
81-90	21656	32.6	8778	30.1	40.5	0.72*	0.63-0.82
91-100	6531	9.8	1979	6.8	30.3	0.50*	0.43-0.57
>100	226	0.3	38	0.1	16.8	0.28*	0.19-0.40
Family members							
Living alone	50040	75.3	21361	73.1	42.7	1	
1	11049	16.6	5182	17.7	46.9	1.11*	1.06-1.17
2	3517	5.3	1695	5.8	48.2	1.10*	1.02-1.19
3	1222	1.8	634	2.2	51.9	1.24*	1.10-1.41
4	350	0.5	189	0.6	54.0	1.32*	1.05-1.66
>4	255	0.4	148	0.5	58.0	1.70*	1.30-2.25
Proponent of referral to IHC							
General practitioner	45990	69.2	19749	67.6	42.9	1	
Hospital	10120	15.2	4774	16.3	47.2	1.27*	1.21-1.33
Residential facilities/other settings	10323	15.5	4686	16.0	45.4	1.08*	1.03-1.13
Prevalent disorder at IHC enrolment							
Mental disorders	2910	4.4	965	3.3	33.2	1	
Neoplasms	16966	25.5	8595	29.4	50.7	1.91*	1.74-2.09
Cardiovascular diseases	9914	14.9	4483	15.3	45.2	1.43*	1.30-1.57
Neurological disorders	7177	10.8	3160	10.8	44.0	1.06	0.96-1.17
Trauma and injury	5453	8.2	1752	6.0	32.1	0.82*	0.74-0.90
Endocrine and metabolic diseases	3904	5.9	1610	5.5	41.2	1.35*	1.21-1.50
Respiratory diseases	3230	4.9	1191	4.1	36.9	1.17*	1.04-1.31
Skin diseases	3177	4.8	1270	4.3	40.0	1.09	0.98-1.23
Musculoskeletal diseases	2382	3.6	819	2.8	34.4	0.87*	0.77-0.99
Other	8665	13.0	4190	14.3	48.4	1.67*	1.52-1.84
Missing	2655	4.0	1174	4.0	44.2	-	-
Duration of IHC event							
≤100	38215	57.5	11929	40.8	31.2	1	
101-200	11306	17.0	6078	20.8	53.8	2.66*	2.54-2.78
201-300	5498	8.3	3538	12.1	64.4	4.12*	3.88-4.38
301-400	8328	12.5	5256	18.0	63.1	4.00*	3.81-4.22
>400	3086	4.6	2408	8.2	78.0	8.22*	7.52-9.00
Travel distance to ED (min)							
<5	24738	37.2	11287	38.6	45.7	1	
6-20	40059	60.3	17310	59.2	43.0	0.87*	0.84-0.90
> 20	964	1.5	351	1.2	36.8	0.79*	0.98-0.92
Missing	672	1.0	261	0.9	45.0	-	-
Time of arrival in ED							
6 am-2 pm			15109	51.7			
2 pm-8 pm			8975	30.7			
8 pm-12 pm			2532	8.7			
12 pm-6 am			2593	8.9			
Destination after discharge from ED							
Home following treatment in ED			15053	51.5			
Admitted to hospital			13056	44.7			
Dead in ED			825	2.8			
Home with no treatment needed in ED			233	0.8			
Residential facilities			42	0.1			
ED triage codes							
Low level of emergency			17569	60.1			
Medium level of emergency			10158	34.8			
High level of emergency			1482	5.1			

Abbreviations: IHC, integrated home care; ED, emergency department; ORs, odds ratios. ^a^ OR of one or more ED visits, computed by logistic regression analysis. All OR were mutually adjusted for any independent variable. * Statistically significant results with a *P* <.05.

 Prevalent disorder at IHC enrolment were neoplasms (25.5%), cardiovascular diseases (14.9%), and neurological disorders (10.8%). Most IHC events (57.5%) were carried out for patients that had a duration of IHC event of ≤100 days.

###  Emergency Department Visits

 Of the 66433 IHC events, 29209 (44 every 100 IHC) had at least one ED visit. The arrival time pattern showed that the peak hours of arrival were mainly during the day (51.7% at 6 am-2 pm; 30.7% at 2 pm-8 pm). In 50.3% of ED visits, the place of discharge was the patient’s home. ED triage codes showed that low levels of emergency were most prevalent (60.1%), with higher level of emergency for patients with a hospital admission (6686 high codes vs. 6170 low codes) compared to patients discharged to their home (4068 high codes vs. 11228 low codes). Patients who died in the ED were admitted with higher emergency codes, with a ratio of more than 4.3 (670 vs. 155).

Analysis showed an increased risk of ED visits in IHC events involving males (OR = 1.29, 95% CI: 1.24, 1.33), patients without a non-family caregiver (OR = 1.13, 95% CI: 1.06-1.22), and patients living with one or more family members (Table 2). Longer travel distances from the patient’s residence to the ED (hospital) reduced the likelihood of an ED visit.

 Referral to IHC by a hospital or a residential facility increased the risk of having an ED visit (OR = 1.27, 95% CI: 1.21-1.33 and OR = 1.08, 95% CI: 1.03-1.13, respectively) when compared to referral by a general practitioner.

 ORs decreased with increasing the age, with the lowest risk of ED visits during IHC events was observed among subjects aged > 100 years (OR = 0.28, 95% CI 0.19-0.40). Additionally, ORs increased with duration of IHC event, being 8.2 times higher (95% CI: 7.52-9.00) for a duration of over 400 days. IHC events among patients affected by neoplasms and cardiovascular diseases showed the highest risk of ED visit (OR = 1.91, 95% CI: 1.74-2.09 and OR = 1.43, 95% CI 1.30-1.57, respectively).

 Figure shows the heatmap of the association between prevalent disorder at IHC enrolment and the pathology registered at the ED visits. ED visits were mainly due to respiratory diseases (acute respiratory insufficiency and pleurisy) and trauma (hip and upper limb fractures). In addition, results showed a correlation between respiratory diseases at ED visit and respiratory diseases or neurological disorders as prevalent disorder at IHC enrolment. There was also a correlation between a diagnosis of trauma at ED admission and trauma and musculoskeletal problems at the first evaluation in IHC.

**Figure:**
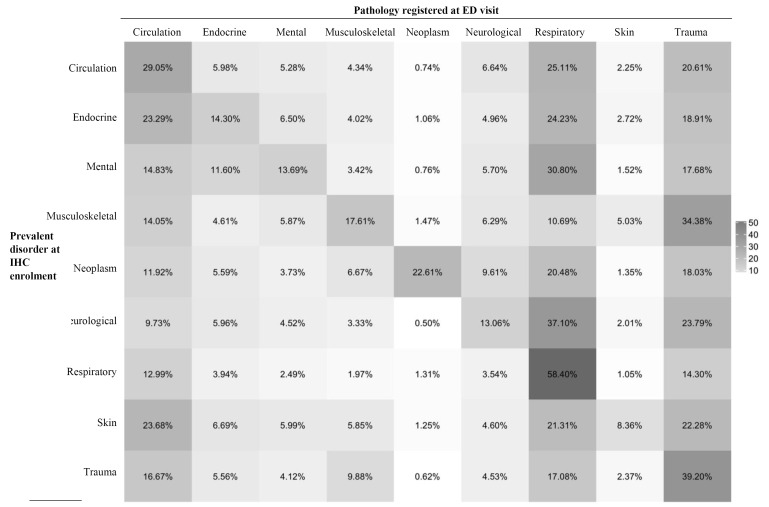
Heatmap Matrix of the associations between prevalent disorder at IHC enrolment and pathology registered at ED visit between 2012 and 2017. Piedmont Region. Abbreviations: IHC, integrated home care; ED, emergency department.

## Discussion

Our results showed significant associations between ED visits and a number of variables, including socio-demographic and clinical variables. For example, chronic disease were shown to be a determinant of ED visits, which is consistent with previous studies in which the chronicity of diseases or severity of illness were found to be the most consistent predictors of ED visits.^16-19^ It is realistic to assume that the increased severity of illness, along with the fragility of patients, which is often associated with the lack of a caregiver, might explain why almost half of the patients were hospitalised after the ED visits, and why ED visits were more frequent for acute exacerbations of pre-existing conditions or for patients referred to IHC by hospitals or residential facilities.

The triage codes we observed are consistent with the instability of patients’ clinical conditions, despite the number of those admitted to hospital with a low emergency triage code. Unfortunately, the triage system, per se, cannot adequately respond to the complexity of the care needs of older patients, particularly when multiple and interrelated conditions are present. The triage system, which has been developed for rapid prioritisation and management of life-threatening conditions, cannot identify these complexities, and a multidimensional evaluation of older patients in the ED is needed, particularly for the frailty condition.^20^ Indeed, underestimation of severity at triage was shown in the 50% of geriatric patients, especially when the cause of the ED visit was trauma.^21^

In contrast to previous studies that reported significantly different risks of ED visits by sex, with fewer ED visits among men,^22,23^ our results showed that men had a higher risk of ED visits. With the available data, it is not possible to assign a valid, exhaustive explanation to these results, and more research is needed to unravel these findings. Another important result of our study was that the highest risk of ED visits was shown for patients who were referred to IHC by hospitals, and that about a half of patients were discharged to their home after ED visits. We can question whether ED visits were related to an inappropriate referral to IHC or whether the ED visit itself was inappropriate. Unfortunately, the current IHC referral processes in Italy still lack some elements that the international literature has described as effective to allow a successful transition from the hospital to the home setting, as Italy does not ensure that patients are discharged at an appropriate time nor does it provide adequate post-discharge services. Because of these coordination and integration problems between hospital and home care services, many patients referred to IHC have frequent ED visits or are readmitted, and then placed back in IHC, experiencing the so-called revolving-door syndrome.^24^

To ensure appropriate referral to IHC, the Italian National Plan of Chronicity^25^ states that an individualised care plan should be developed for the patient prior to leaving the hospital. Unfortunately, a retrospective observational cohort study conducted in the same region, in a sample of 380 subjects aged over 64 years, showed that hospital readmission rates after referral to IHC and the completion of an individualised care plan did not significantly differ from those for a standard hospital discharge. About one in 5 patients (22.1%) was readmitted within 6 months after standard hospital discharge.^26^ Barriers to avoiding inappropriate ED visits or hospital admissions are various, and include variation in frequency of services offered; difficulties in agreeing upon the most appropriate professional to lead the IHC team; and a lack of acute assessment skills.^27^

Specialised multidisciplinary community-based interventions in primary care showed positive outcomes; to be effective, robust home-based programmes should involve fully integrated interprofessional care teams, regular interprofessional care meetings, comprehensive geriatric assessments at enrolment, and an after-hours urgent telephone service.^28^ Limited evidence of avoidance of inappropriate ED visits was indeed produced for tele-health and other electronic system approaches for patients recently discharged from hospitals.^27^

The presence of non-family caregivers can slightly reduce the risk of ED visits; their presence was identified as an effective preventive measure, as they were often trained in monitoring and supervising the patient and are completely devoted to the patient’s care and assistance.^29^ In northern and central Italy, more than half of formal care workers provide basic assistance, 1 in 3 assist people who are not self-sufficient, and at least 1 in 5 is employed in advanced care.^30^

Italy, like other southern European countries, is commonly referred to as a ‘strong-family-ties country.’^31^ Considering that, the observed increase in the risk of ED visits with increasing number of cohabitating family members, together with the reduced risk observed when the patient is reported to live alone, is unexpected. Having to assume family roles (mother/father, son/daughter) and occupational roles in addition to a caregiver role is likely to create role conflicts, which can lead to an increased risk of psychological distress and anxiety for the caregiver.^32-35^ Compared to those with only an occupational role, family caregivers are more exposed to an inability to take accountability for the relative/patient’s healthcare.^36^

Our results showed that, as the distance between the patient’s home and the closest ED increases, the risk of ED visits decreases. Coherently with previous studies in France,^37^ Sweden,^38^and the United Kingdom,^39^a reduced travel distance can increase the likelihood ED visits, independently of the severity of the medical condition^40,41^ thus increasing health costs despite the effective need.

 The findings of this study should be read while taking into account embedded study limitations and strengths. Major limitations are those regarding the information sources used and are common to all administrative database studies. These include problems related to the quality of data collection, especially the possible lack of accuracy and different coding criteria used by individuals and across different institutions. Moreover, data on socio-demographic variables, such as individual education and income level, are often not recorded in administrative databases, and thus caution regarding their reliability is warranted when these variables are present. By contrast, these databases are the best available for large-scale epidemiological morbidity studies and for monitoring population trends in service utilisation. Lastly, given the purpose of administrative databases, it is difficult to determine the quality of delivered care, as it only possible to indicate definite groups of patients to which a specific intervention was targeted to reduce the risk of an ED visit during an IHC event. It is also proper to mention that the lack of a control group is a relevant limitation of the current study.

Among the main strengths of the study are the fact that it deals with an issue that is common to all public healthcare systems, as they are called to shift from hospital care to home care and to provide assistance that is appropriate to the specific health need in any setting. Consequently, the evidence of socio-demographic and clinical determinants of ED visits may offer home care service providers a useful perspective to implement intervention programmes based both on appropriate individual care plans and broad-based client assessments. Moreover, all results could be helpful in other European countries, as the study was based in a large northern Italian region that has healthcare systems and population health profiles comparable to those of other European countries.^42^

## Ethical issues

 Data were merged using the universal patient ID number, within each observation year, to ensure that specific ED visits were linked to the appropriate IHC event. The universal patient ID number is an anonymous, unique code assigned to each patient within the ministerial system fluxes in order to guarantee medical record traceability. Once anonymised, available sources are made available to identified academic or research institutions for administrative and/or epidemiological studies without any further authorisation. Therefore, ethics committee approval was not required and it was not necessary.

## Competing interests

 Authors declare that they have no competing interests.

## Authors’ contributions

 MMG formulated the research goals and supervised the research activity. MMG, SC, and GP defined the design of the methodology. SC, MMG, and ABo wrote the article. MMG, ABo and VD revised the article. GP and ABe planned and performed statistical analyses. MD collected the data and managed the database.

## Authors’ affiliations

^1^Department of Public Health and Paediatrics, Università di Torino, Torino, Italy. ^2^Department of Control and Computer Engineering, Politecnico di Torino, Torino, Italy. ^3^Epidemiology Unit, Local Health Unit TO3, Grugliasco (TO), Piedmont, Italy.

## Supplementary files

Supplementary file 1. Risk Analysis POP.1.
