# Supplementary material for: Determinants Associated With the Risk of Emergency Department Visits Among Patients Receiving Integrated Home Care Services: A 6-Year Retrospective Observational Study in a Large Italian Region
Source: Int J Health Policy Manag. 2020 Jun 7;10(10):605–12. doi: 10.34172/ijhpm.2020.79 (PMC9278539; doi:10.34172/ijhpm.2020.79)

Supplementary file 1. Risk Analysis POP.1

Risk Analysis

EDxIHC vs. SEX

Confusion Matrix Computed

Confusion Matrix (Fisher's Exact Test for Count Data) - **p-value = 6.55e-84**

	EDxIHC +	EDxIHC -	Total	Inc risk *	Odds
**SEX+**	16355	15046	31401	52.1	1.09
**SEX-**	20869	14163	35032	59.6	1.47
**Total**	37224	29209	66433	56.0	1.27

Risk-Analysis Results

X2 test statistic - **p-value: 0**

dati	Estimate	95%CI +	95%CI -
**Risk Ratio**	0.874	0.862	0.886
**Odds Ratio**	0.738	0.715	0.761
**Attr.Risk**	-7.49	-8.24	-6.73
**Pop.Attr.Risk**	-3.54	-4.18	-2.9
**Attr.Fract.Risk**	-0.144	-0.159	-0.128
**Pop.Attr.Fract.Risk**	-0.063	-0.07	-0.057

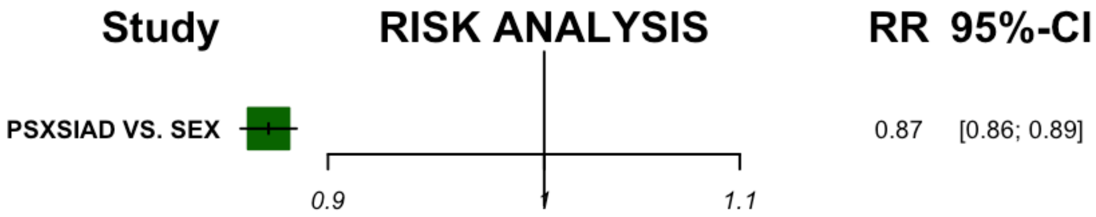

EDxIHC vs. ASS_FAM

ASS_FAM= Presence of a non-family caregiver

Confusion Matrix Computed

Confusion Matrix (Fisher's Exact Test for Count Data) - **p-value = 5.44e-06**

	EDxIHC +	EDxIHC -	Total	Inc risk *	Odds
**ASS_FAM+**	2720	1871	4591	59.2	1.45
**ASS_FAM-**	34504	27338	61842	55.8	1.26
**Total**	37224	29209	66433	56.0	1.27

Risk-Analysis Results

X2 test statistic - **p-value: 0**

dati	Estimate	95%CI +	95%CI -
**Risk Ratio**	1.06	1.04	1.09
**Odds Ratio**	1.15	1.08	1.22

dati	Estimate	95%CI +	95%CI -
**Attr.Risk**	3.45	1.98	4.93
**Pop.Attr.Risk**	0.239	-0.305	0.782
**Attr.Fract.Risk**	0.058	0.034	0.082
**Pop.Attr.Fract.Risk**	0.004	0.002	0.006

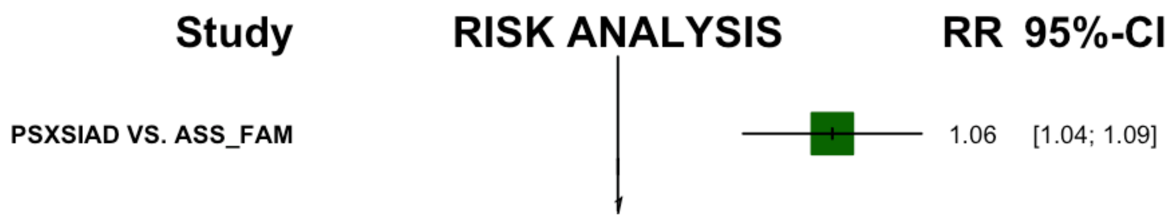

Risk Analysis

AGE vs. EDxIHC

Show/Hide Details

VAL-GROUP Confusion Matrix

Fisher's Exact Test for Count Data, **p: 9.76e-20**
RR:0.784565916398714

	EDxIHC 1	EDxIHC 0	IR%
**Age 19-65**	6840	7187	0.488
**Ref. <=18**	769	467	0.622

Fisher's Exact Test for Count Data, **p: 4.75e-24**
RR:0.763665594855305

	EDxIHC 1	EDxIHC 0	IR%
**Age 66-80**	10805	11952	0.475
**Ref. <=18**	769	467	0.622

Fisher's Exact Test for Count Data, **p: 3.97e-50**
RR:0.65112540192926

	EDxIHC 1	EDxIHC 0	IR%
**Age 81-90**	8778	12878	0.405
**Ref. <=18**	769	467	0.622

Fisher's Exact Test for Count Data, **p: 3.70e-98**
RR:0.487138263665595

	EDxIHC 1	EDxIHC 0	IR%
**Age 91-100**	1979	4552	0.303
**Ref. <=18**	769	467	0.622

Fisher's Exact Test for Count Data, **p: 7.83e-38**
RR:0.270096463022508

	EDxIHC 1	EDxIHC 0	IR%
**Age >100**	38	188	0.168
**Ref. <=18**	769	467	0.622

MULTI_GROUP Confusion Matrix

	EDxIHC +	EDxIHC -	Total	Inc risk *	Odds
**Age +**	28440	36757	65197	43.6	0.774
**Age -**	3845	2335	6180	62.2	1.647
**Total**	32285	39092	71377	45.2	0.826

Risk-Analysis Results

X2 test statistic - **p-value: 0**

dati	est	lower	upper
**Risk Ratio**	0.659	0.643	0.676
**Odds Ratio**	0.422	0.398	0.448
**Attr.Risk**	-21.2	-27	-15.3
**Pop.Attr.Risk**	-17	-18.2	-15.7
**Attr.Fract.Risk**	-0.426	-0.457	-0.396
**Pop.Attr.Fract.Risk**	-0.376	-0.391	-0.359

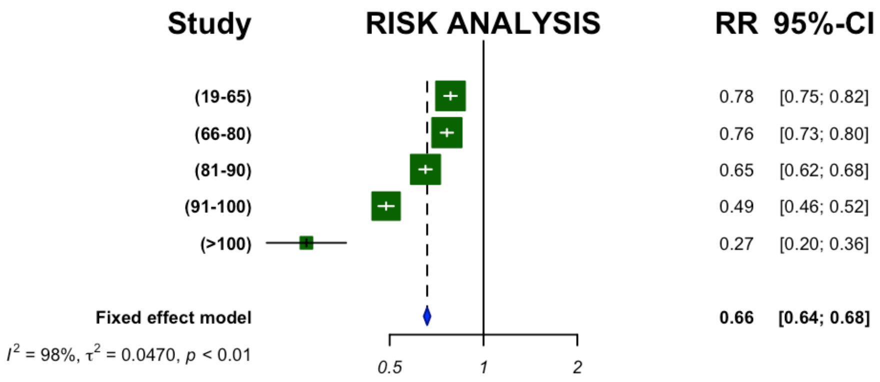

FAMILY MEMBERS vs. EDxIHC

Show/Hide Details

VAL-GROUP Confusion Matrix

Fisher's Exact Test for Count Data, **p: 7.58e-16**
RR:1.0983606557377

	EDxIHC 1	EDxIHC 0	IR%
**Family members 1_pers_**	5182	5867	0.469

	EDxIHC 1	EDxIHC 0	IR%
**Ref. 0 pers.**	21361	28679	0.427

Fisher's Exact Test for Count Data, **p: 2.20e-10**
RR:1.1288056206089

	EDxIHC 1	EDxIHC 0	IR%
**Family members 2_pers_**	1695	1822	0.482
**Ref. 0 pers.**	21361	28679	0.427

Fisher's Exact Test for Count Data, **p: 1.97e-10**
RR:1.21545667447307

	EDxIHC 1	EDxIHC 0	IR%
**Family members 3_pers_**	634	588	0.519
**Ref. 0 pers.**	21361	28679	0.427

Fisher's Exact Test for Count Data, **p: 2.78e-05**
RR:1.26463700234192

	EDxIHC 1	EDxIHC 0	IR%
**Family members 4_pers_**	189	161	0.54

	EDxIHC 1	EDxIHC 0	IR%
**Ref. 0 pers.**	21361	28679	0.427

Fisher's Exact Test for Count Data, **p: 1.18e-06**
RR:1.35831381733021

	EDxIHC 1	EDxIHC 0	IR%
**Family members 4+*pers***	148	107	0.58
**Ref. 0 pers.**	21361	28679	0.427

MULTI_GROUP Confusion Matrix

	EDxIHC +	EDxIHC -	Total	Inc risk *	Odds
**Family members +**	7848	8545	16393	47.9	0.918
**Family members -**	106805	143395	250200	42.7	0.745
**Total**	114653	151940	266593	43.0	0.755

Risk-Analysis Results

X2 test statistic - **p-value: 0**

dati	est	lower	upper
**Risk Ratio**	1.12	1.1	1.14
**Odds Ratio**	1.24	1.2	1.28

dati	est	lower	upper
**Attr.Risk**	5.31	1.71	8.9
**Pop.Attr.Risk**	0.319	0.049	0.589
**Attr.Fract.Risk**	0.108	0.093	0.123
**Pop.Attr.Fract.Risk**	0.007	0.007	0.008

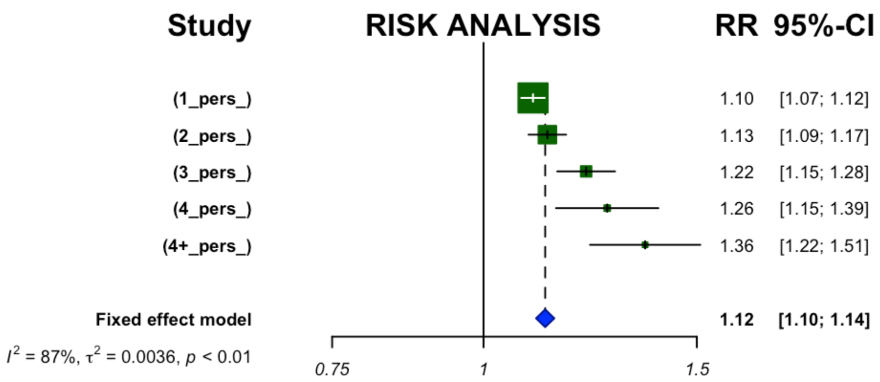

PAT_PREV vs. EDxIHC

PAT_PREV = **Prevalent disorder at IHC enrolment**

Show/Hide Details

VAL-GROUP Confusion Matrix

Fisher's Exact Test for Count Data, **p: 4.90e-04**
RR:0.954635108481262

	EDxIHC 1	EDxIHC 0	IR%
**PAT_PREV Altro**	4190	4475	0.484
**Ref. Neoplasm**	8595	8371	0.507

Fisher's Exact Test for Count Data, **p: 7.05e-18**
RR:0.891518737672584

	EDxIHC 1	EDxIHC D 0	IR%
**PAT_PREV Circulatory**	4483	5431	0.452
**Ref. Neoplasm**	8595	8371	0.507

Fisher's Exact Test for Count Data, **p: 2.07e-26**
RR:0.812623274161736

	EDxIHC 1	EDxIHC 0	IR%
**PAT_PREV Endocrine**	1610	2294	0.412
**Ref. Neoplasm**	8595	8371	0.507

Fisher's Exact Test for Count Data, **p: 1.66e-69**
RR:0.654832347140039

	EDxIHC 1	EDxIHC 0	IR%
**PAT_PREV Mental**	965	1945	0.332
**Ref. Neoplasm**	8595	8371	0.507

Fisher's Exact Test for Count Data, **p: 7.17e-10**
RR:0.871794871794872

	EDxIHC 1	EDxIHC 0	IR%
**PAT_PREV Missing**	1174	1481	0.442
**Ref. Neoplasm**	8595	8371	0.507

Fisher's Exact Test for Count Data, **p: 7.70e-51**
RR:0.678500986193294

	EDxIHC 1	EDxIHC 0	IR%
**PAT_PREV MuscoloSkeletal**	819	1563	0.344
**Ref. Neoplasm**	8595	8371	0.507

Fisher's Exact Test for Count Data, **p: 4.18e-21**
RR:0.867850098619329

	EDxIHC 1	EDxIHC 0	IR%
**PAT_PREV Nervous**	3160	4017	0.44

	EDxIHC 1	EDxIHC 0	IR%
**Ref. Neoplasm**	8595	8371	0.507

Fisher's Exact Test for Count Data, **p: 2.86e-47**
RR:0.727810650887574

	EDxIHC 1	EDxIHC 0	IR%
**PAT_PREV Respiratory**	1191	2039	0.369
**Ref. Neoplasm**	8595	8371	0.507

Fisher's Exact Test for Count Data, **p: 1.49e-28**
RR:0.788954635108481

	EDxIHC 1	EDxIHC 0	IR%
**PAT_PREV Skin**	1270	1907	0.4
**Ref. Neoplasm**	8595	8371	0.507

Fisher's Exact Test for Count Data, **p: 1.34e-128**
RR:0.633136094674556

	EDxIHC 1	EDxIHC 0	IR%
**PAT_PREV Trauma**	1752	3701	0.321
**Ref. Neoplasm**	8595	8371	0.507

MULTI_GROUP Confusion Matrix

	EDxIHC +	EDxIHC -	Total	Inc risk *	Odds
**PAT_PREV+**	20614	28853	49467	41.7	0.714
**PAT_PREV-**	85950	83710	169660	50.7	1.027
**Total**	106564	112563	219127	48.6	0.947

Risk-Analysis Results

X2 test statistic - **p-value: 0**

dati	est	lower	upper
**Risk Ratio**	0.814	0.804	0.823
**Odds Ratio**	0.683	0.669	0.697
**Attr.Risk**	-9.44	-10.2	-8.65
**Pop.Attr.Risk**	-2.03	-2.35	-1.71
**Attr.Fract.Risk**	-0.216	-0.23	-0.202
**Pop.Attr.Fract.Risk**	-0.042	-0.042	-0.041

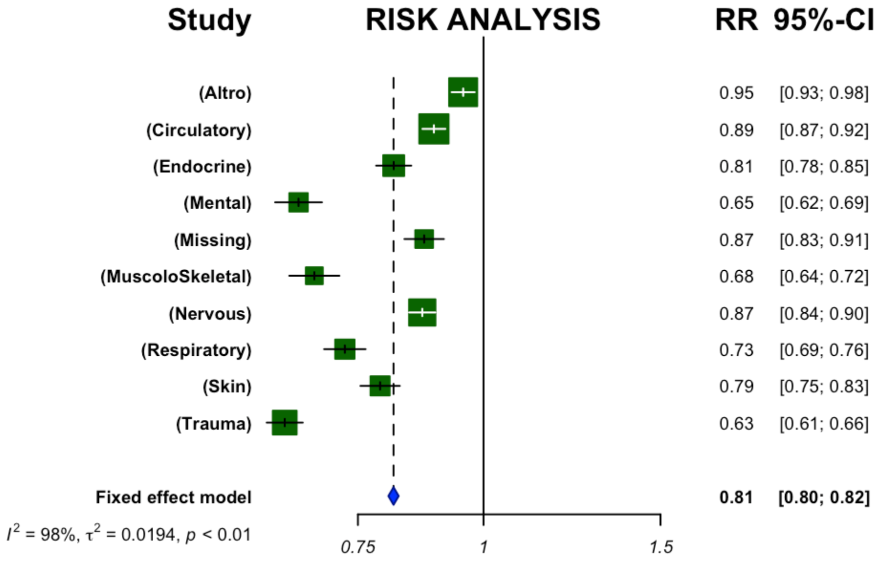

DURATION OF IHC EVENT vs. EDxIHC

GA= Days

Show/Hide Details

VAL-GROUP Confusion Matrix

Fisher's Exact Test for Count Data, **p: 0.00e+00**
RR:1.72435897435897

	EDxIHC 1	EDxIHC 0	IR%
**GA_101-200**	6078	5228	0.538
**Ref. GA.<100**	11929	26286	0.312

Fisher's Exact Test for Count Data, **p: 0.00e+00**
RR:2.06410256410256

	EDxIHC 1	EDxIHC 0	IR%
**GA_201-300**	3538	1960	0.644
**Ref. GA.<100**	11929	26286	0.312

Fisher's Exact Test for Count Data, **p: 0.00e+00**
RR:2.0224358974359

	EDxIHC 1	EDxIHC 0	IR%
**GA_301-400**	5256	3072	0.631
**Ref. GA.<100**	11929	26286	0.312

Fisher's Exact Test for Count Data, **p: 0.00e+00**
RR:2.5

	EDxIHC 1	EDxIHC 0	IR%
**GA_>400**	2408	678	0.78
**Ref. GA.<100**	11929	26286	0.312

MULTI_GROUP Confusion Matrix

	EDxIHC +	EDxIHC -	Total	Inc risk *	Odds
**GA+**	17280	10938	28218	61.2	1.580

	EDxIHC +	EDxIHC -	Total	Inc risk *	Odds
**GA-**	47716	105144	152860	31.2	0.454
**Total**	64996	116082	181078	35.9	0.560

Risk-Analysis Results

X2 test statistic - **p-value: 0**

dati	est	lower	upper
**Risk Ratio**	1.98	1.95	2
**Odds Ratio**	3.55	3.45	3.64
**Attr.Risk**	30.5	28.6	32.4
**Pop.Attr.Risk**	4.68	4.36	5
**Attr.Fract.Risk**	0.49	0.484	0.496
**Pop.Attr.Fract.Risk**	0.13	0.129	0.131

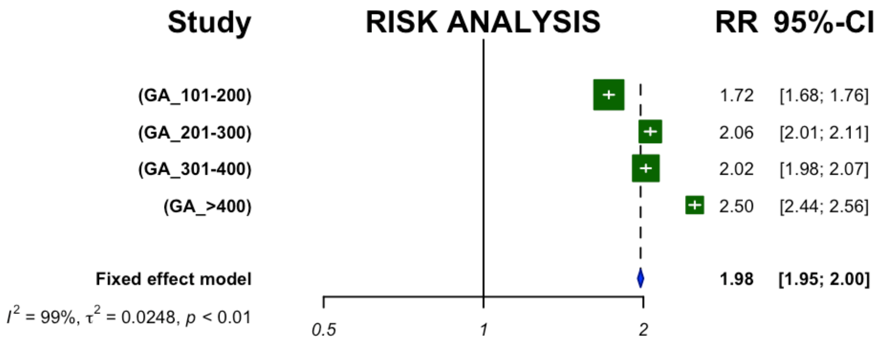

PROPONENT of referral to IHC vs. EDxIHC

PROP_ PA_02+06+08+09=Residential facilities/other settings

PROP_ PA.01= General practitioner

PROP_ PA_03-04-05-07= Hospital

Show/Hide Details

VAL-GROUP Confusion Matrix

Fisher's Exact Test for Count Data, **p: 5.97e-06**
RR:1.05827505827506

	EDxIHC 1	EDxIHC 0	IR%
**PROP_ PA_02+06+08+09**	4686	5637	0.454
**Ref. PA.01**	19749	26241	0.429

Fisher's Exact Test for Count Data, **p: 9.18e-15**
RR:1.1002331002331

	EDxIHC 1	EDxIHC 0	IR%
**PROP PA_03-04-05-07**	4774	5346	0.472
**Ref. PA.01**	19749	26241	0.429

MULTI_GROUP Confusion Matrix

	EDxIHC +	EDxIHC -	Total	Inc risk *	Odds
**PROP+**	9460	10983	20443	46.3	0.861
**PROP-**	39498	52482	91980	42.9	0.753
**Total**	48958	63465	112423	43.5	0.771

Risk-Analysis Results

X2 test statistic - **p-value: 0**

dati	est	lower	upper
**Risk Ratio**	1.08	1.06	1.1
**Odds Ratio**	1.15	1.11	1.18
**Attr.Risk**	3.33	1.83	4.84
**Pop.Attr.Risk**	0.606	0.174	1.04
**Attr.Fract.Risk**	0.072	0.056	0.087
**Pop.Attr.Fract.Risk**	0.014	0.013	0.015

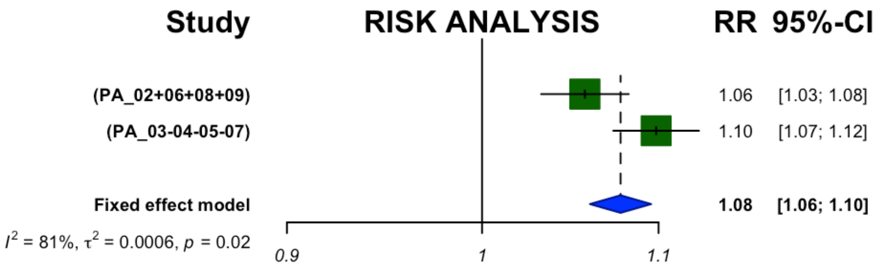

Supplement: Supplementary file 1 — Risk Analysis POP.1. [file ijhpm-10-605-s001.pdf]
